# Novel adenoviral serotype 5 based immunotherapeutic induces T-cell responses despite anti-adenoviral neutralizing antibodies in colorectal cancer patients

**DOI:** 10.1186/2051-1426-1-S1-P129

**Published:** 2013-11-07

**Authors:** Michael Morse, Elizabeth Gabitzsch, Joseph P Balint, Younong Xu, HK Lyerly, Frank Jones

**Affiliations:** 1 Etubics Corporation, Seattle, WA, USA; 2 Duke University Medical Center, Durham, NC, USA

## 

Naturally occurring or induced Ad-specific neutralizing antibodies impeded the activity of recombinant Ad5-based vectors with E1 deletions. An improved Ad vector with deletions of the E1 and the E2b regions (Ad5 [E1-, E2b-]), the latter encoding the DNA polymerase and the pre-terminal protein, with significantly diminished late phase viral protein expression, were hypothesized to avoid immunological clearance and induce more potent immune responses against the encoded tumor antigen transgene in Ad-immune hosts. In the present phase I/II study, cohorts of patients with advanced metastatic colorectal cancer (mCRC) were immunized with escalating doses of Ad5 [E1-, E2b-]-CEA(6D). The carcinoembryonic antigen (CEA) transgene employed contains a modification (CAP1-6D) designed to enhance CTL stimulation. CEA-specific CMI responses were observed despite the presence of pre-existing Ad5 immunity in a majority (61.3%) of patients. Long-term follow-up in some of the patients reveled a waning of the induced CEA directed immune responses at around 6 months. Importantly, there was minimal toxicity, and overall patient survival (48% at 12 months) was similar regardless of pre-existing Ad5 neutralizing antibody titers. Our patient demographics, albeit limited in size, we similar with previously published studies of patients with chemotherapy-refractory mCRC. Of particular interest is the observation that treated mCRC patients in our study exhibited favorable survival probability. The results demonstrate that, in cancer patients, the novel Ad5 [E1-, E2b-] gene delivery platform generates significant CMI responses to the tumor antigen CEA in the setting of both naturally acquired and immunization-induced Ad5-specific immunity.

